# Oxalic Acid-Induced Photodissolution of Ferrihydrite and the Fate of Loaded As(V): Kinetics and Mechanism

**DOI:** 10.3390/nano9081143

**Published:** 2019-08-09

**Authors:** Hai-Tao Ren, Jing Han, Ting-Ting Li, Qi Lin, Jia-Horng Lin, Ching-Wen Lou

**Affiliations:** 1 Innovation Platform of Intelligent and Energy-Saving Textiles, School of Textile Science and Engineering, Tianjin Polytechnic University, Tianjin 300387, China; 2 Tianjin and Ministry of Education Key Laboratory for Advanced Textile Composite Materials, Tianjin Polytechnic University, Tianjin 300387, China; 3 Fujian Key Laboratory of Novel Functional Fibers and Materials, Minjiang University, Fuzhou 350108, China; 4 Fujian Engineering Research Center of New Chinese Lacquer Material, Minjiang University, Fuzhou 350108, China; 5 School of Chinese Medicine, China Medical University, Taichung 40402, Taiwan; 6 Laboratory of Fiber Application and Manufacturing, Department of Fiber and Composite Materials, Feng Chia University, Taichung 40724, Taiwan; 7 Ocean College, Minjiang University, Fuzhou 350108, China; 8 College of Textile and Clothing, Qingdao University, Qingdao 266071, China; 9 Department of Fashion Design, Asia University, Taichung 41354, Taiwan; 10 Department of Bioinformatics and Medical Engineering, Asia University, Taichung 41354, Taiwan; 11 Department of Medical Research, China Medical University Hospital, China Medical University, Taichung 40402, Taiwan

**Keywords:** ferrihydrite, As(V), photodissolution, oxalic acid, fate

## Abstract

The fate of arsenic in the water environment is of great concern. Here, the influences of oxalic acid and UV light illumination on the dissolution of naked ferrihydrite (Fhy), Fhy loaded with As(V) [Fhy*-As(V)], as well as the fate of As(V) at pH 3.0 were studied. With the assistance of oxalic acid, complexes of Fe(III)-oxalic acid produced on Fhy/Fhy*-As(V) were reduced to Fe(II)-oxalic acid by photo-induced electrons under UV light irradiation. UV light has nearly no impact on the release of As(V) in the system of Fhy*-As(V) without the assistance of oxalic acid. Nevertheless, in the existence of oxalic acid, UV light illumination resulted in the contents of liberated As(V) decreased by 775–1300% compared to that without light. Considering the coexistence of As(V), oxalic acid as well as iron oxides in aquatic environments, the present study revealed that UV illumination could enhance the retention of As(V) on Fhy in the acidic water environment containing oxalic acid.

## 1. Introduction

In aquatic environments, arsenic (As) is an omnipresent toxic pollutant and mainly occurs as arsenite [As(III)] and arsenate [As(V)] with inorganic forms [1]. It has been reported that cardiovascular diseases and kidney, skin, liver, bladder, and lung cancers are associated with chronic exposure to As [2,3]. The presence of As in drinking water is a result of the migration of As always caused by weathering of As-bearing minerals, geochemical reactions, volcanic emissions, microbial processes, and other anthropogenic factors containing waste discharge, mining, and use of corresponding pesticides [4]. On account of the serious toxicity of As, the United States Environmental Protection Agency (USEPA) in 2001 lowered the guideline from 50 to 10 μg L^−1^ for total As in drinking water [5]. To attain this stringent threshold, several technologies have been used to remove As from water, and these contain coagulation–flocculation [6], precipitation [6], ion exchange [6], membrane separation [6], electrochemical methods [6], photocatalysis [7,8], and adsorption [9,10]. Out of these techniques, adsorption is considered one of the most promising techniques due to its low processing cost, simple operation, and high efficiency at different pH of water [6,10].

Ferric (oxyhydr)oxides are considered as the most important adsorbents for fixing As due to their high natural abundance and good binding affinities to As [11,12,13]. It is obvious that the migration of As is dominantly regulated by the dissolution of ferric (oxyhydr)oxides [14]. A previous study demonstrated that ferric (oxyhydr)oxides could be reduced by Fe(III)-reducing bacteria, which led to the dissolution and subsequent liberation of As fixed on those Fe minerals [15]. Additionally, abiotic factors which triggered dissolution of ferric (oxyhydr)oxides makes a contribution to As migration.

In general, photo-triggered reductive dissolution of ferric (oxyhydr)oxides contains two key reaction stages. Firstly, Fe(III) on the (oxyhydr)oxides surface is photoreduced to Fe(II). Secondly, the formed surface Fe(II) is liberated into solution, which determines the overall photodissolution rate [16,17,18,19,20]. Two different mechanisms have been verified to explain the emergence of surface Fe(II) in photic water environments: (a) UV light-triggered charge transfer between lattice O(-II) and Fe(III) in ferric (hydr)oxides produces holes and electrons, and then the latter will cause the reduction of surface Fe(III); (b) the production of surface Fe(II) derives from LMCT (ligand-to-metal charge transfer) reaction in light-reactive surface Fe(III)-organic acid complexes [18]. Dissolved organic matters (DOM) could significantly improve the light-triggered dissolution rate of ferric (hydr)oxides [16,18,19,20]. For example, Borer et al. [20] have found that siderophores [aerobactin and desferrioxamine B (DFOB)] accelerated the light-triggered dissolution rate of lepidocrocite (γ-FeOOH), and the value increased 91-fold at pH 3.0 with the assistance of DFOB [20].

Oxalic acid is widely distributed in natural water and exhibits a prominent contribution to the dissolution of minerals containing Fe [21]. Considerable attention has been paid to the irradiated oxalic acid–iron (hydr)oxide systems in nature, because pollutants can be degraded via the photo-assistant Fenton-like reaction in such systems [22,23,24,25,26]. Wu et al. [26] revealed that at pH 4.0 dissolved Fe(II) with concentration of 25.0 mg L^−1^ was yielded by UV illumination for 5 min in the system of oxalate (2.0 mM) and schwertmannite (0.2 g L^−1^). After 40 min of UV illumination, the removal percentage of 50.0 mg L^−1^ methyl orange was 97%. Nevertheless, very limited attention has been focused on photo-assistant chemical interactions of the ternary ferric (oxyhydr)oxides-oxalic acid-As(V) systems. Meanwhile, under UV irradiation, the impact of oxalic acid on the fate of As(V) fixed on ferric (oxyhydr)oxides has not been thoroughly examined, and the corresponding mechanism for retention of the liberated As(V) by the residual or newly-produced minerals needs to be fully comprehended.

Ferrihydrite (Fhy, Fe_5_HO_8_·4H_2_O) is an amorphous to semiamorphous iron mineral mainly ranging in size from 3.0 to 10.0 nm. It is omnipresent in nature and has been recognized as one of the most important adsorbents for anions. Our previous study suggested that Fhy has an excellent adsorption capacity for As(V) (160 mg g^−1^), owing to its larger specific surface area and higher density of reactive hydroxyl groups [27]. Herein, the present study focused on understanding the impacts of oxalic acid and fixed As(V) amount on the light-triggered dissolution of Fhy, and the mechanism of As(V) migration involved in this photodissolution process.

## 2. Materials and Methods

### 2.1. Synthesis of Fhy and Fhy*-As(V)

Fhy used in the present work was the 2-line ferrihydrite, and it was prepared by applying the method developed by Cornell and Schwartzman [12]. In a typical synthesis, twenty gram of Fe(NO_3_)_3_·9H_2_O (Aladdin Chemicals, Shanghai, China) was dispersed in 250 mL deionized water and then 1.0 M NaOH (Aladdin Chemicals, Shanghai, China) was introduced into the solution by vigorous stirring at room temperature to maintain the pH at 7.0. Afterwards, the suspension was aged for 12 h under the static condition. The resulting brown precipitates were concentrated by centrifuging and washed repeatedly with deionized water for four times.

In terms of our previous study [27], for the synthesis of Fhy loaded with As(V), the corresponding adsorption kinetics were operated at pH 3.0. NaOH (0.1 M) and HCl (Aladdin Chemicals, Shanghai, China) (0.1 M) solution were employed to adjust the pH. Specifically, Fhy was firstly dispersed in NaCl (Aladdin Chemicals, Shanghai, China) solution (0.1 M, 100 mL) at pH 3.0 for 24 h to achieve adsorption equilibrium. Afterwards, NaCl solution (0.1 M, 50 mL) including predetermined contents of As(V) with pH 3.0 was blended intensively with the above Fhy suspension. Initial concentration of Fhy in the obtained suspensions was 2.0 g L^−1^, and the respective concentrations of As(V) were 200, 300, and 500 mg L^−1^. After shaking continuously for 24 h with speed of 150 rpm on a shaker, the mixture was centrifuged for 10 min at 4200 rpm, and then washed four times with distilled water. The obtained samples [Fhy loaded with As(V)] were labeled as Fhy*-As(V). In addition, the collected supernate after centrifugation was employed to measure the residual aqueous As(V) contents. Apart from characterization analysis (the obtained solid was freeze dried), the synthesized Fhy and Fhy*-As(V) were directly stored in deionized water.

### 2.2. Photodissolution Kinetics

In this study, dissolution kinetics were conducted in a Model XPA-VII photocatalytic apparatus (Xujiang Electromechanical Plant, Nanjing, China). During the experimental process, the 300 W Hg lamp was turned on 5 min in advance to provide stable illumination intensity. Fhy was firstly dispersed in NaCl solution (0.1 M, 15 mL) with pH 3.0. To investigate the dissolution of Fhy, oxalic acid in NaCl solution (0.1 M, 15 mL) with equal pHs was mingled with the above suspension. The respective concentration of oxalic acid and Fhy was 1.0 mM and 0.1 g L^−1^. The obtained suspensions were continuously agitated at a speed of 800 ± 10 rpm with a magnetic stirrer during the dissolution process. Circulating cooling water was employed to provide a stationary temperature of 25 ± 1 °C. For an overall comparison, photo-triggered dissolution kinetics of Fhy and Fhy*-As(V) were carried out as well without the assistance of oxalic acid. During the dissolution process, 1.0 mL samples were taken regularly and centrifuged in 1.5 mL Eppendorf tubes (12,000 rpm, 5 min), and the obtained supernatant was applied to detect the concentrations of aqueous Fe(II), total Fe (denoted as TFe) and As(V). All kinetics were repeated twice and the average data were recorded. 

### 2.3. Solution Analysis and Characterization Methods

The modified molybdate-blue method [28], with the detection limit of 0.005 mg L^−1^, was employed to determine the concentration of aqueous As(V). Contents of aqueous Fe(II) and TFe were determined by an improved ferrozine method [29]. X-ray diffraction (XRD; D8 Discover, Bruker, Germany) patterns of ferric (hydr)oxides were analyzed with Cu*K_a_* radiation (λ = 1.5406 Å). The morphology of Fhy was analyzed using a high-resolution transmission electron microscopy (HRTEM; Tecnai G2F20, FEI, Hillsboro, OR, USA) before and after the dissolution process. The Fourier transformation infrared spectroscopy (FTIR; Nicolet iS50, Thermo Fisher Scientific, Waltham, MA, USA) was utilized to detect ferric (hydr)oxides.

## 3. Results and Discussion

### 3.1. Characterization of Fhy and Fhy*-As(V)

**Figure 1:**
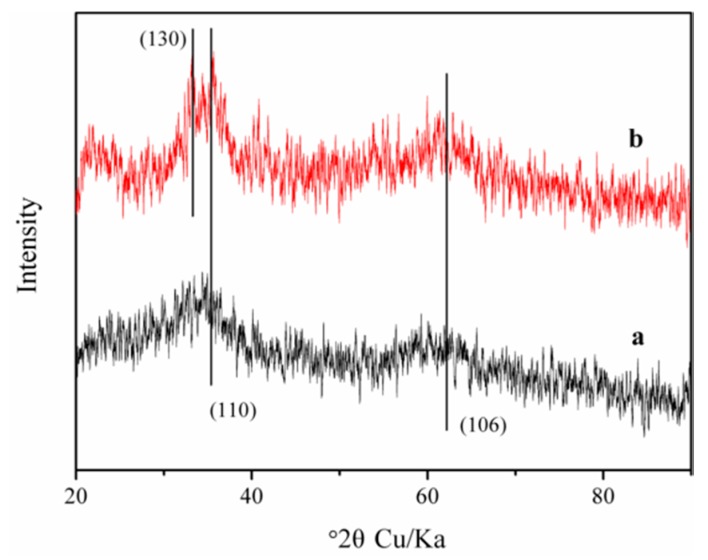
XRD patterns of Fhy (**a**) before and (**b**) after the photodissolution with oxalic acid.

The curve a in Figure 1 manifested the XRD result of the synthetic Fhy, in which all diffraction peaks are in conformity to 2-line Fhy (JCPDS, No. 29-0712). In detail, the respective peak position at 2θ of 35.9° and 62.7° was indexed to the (110) and (106) planes of naked 2-line Fhy [12].

In this study, Fhy*-As(V)-i (i = a, b or c) represented Fhy loaded with different amounts of As(V). Results shown in Table 1 suggested that the respective contents of As(V) over Fhy surface were 99.9, 146.8, and 196.8 mg g^−1^ for Fhy*-As(V)-a, Fhy*-As(V)-b, and Fhy*-As(V)-c. Raven et al. [11] demonstrated that adsorption maxima of 0.25 mol_As(V)_ mol_Fe_^−1^ was achieved by Fhy at pH 4.6.

### 3.2. Effect of Oxalic Acid on the Photodissolution of Fhy

**Figure 2:**
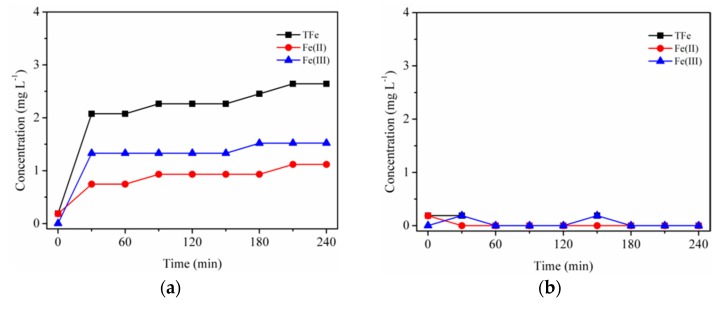
Dissolution kinetics with suspensions of 0.1 g L^−1^ Fhy (**a**) under UV illumination and (**b**) in the dark at pH 3.0 without oxalic acid.

Without the existence of oxalic acid, the dissolution of naked Fhy was investigated in the dark or under UV illumination, and contents of aqueous Fe(II), Fe(III), and TFe were showed in Figure 2. Aqueous TFe and Fe(II) in Fhy suspension increased at the beginning stage and then leveled off at 2.07 and 0.93 mg L^−1^ after 4 h of UV illumination, respectively (Figure 2a). Nevertheless, it is observed from Figure 2b that Fhy dissolution only caused aqueous TFe of 0.18 mg L^−1^, without the occurrence of aqueous Fe(II) in the dark. Without the assistance of oxalic acid, the appearance of aqueous Fe(II) by UV illumination can merely be clarified via the photo-triggered reduction of Fe(III) over the Fhy surface. This further demonstrated that photodissolution of Fhy can occur under acidic conditions (e.g., pH = 3.0) without the assistance of DOM, even though with small reaction rate. Borer et al. [20] indicated that after 360 min of UV illumination of Fhy suspensions with contents of 25 mg L^−1^, 8 μM dissolved TFe was produced at pH 3.0 and 68% of TFe existed as Fe(II).

**Figure 3:**
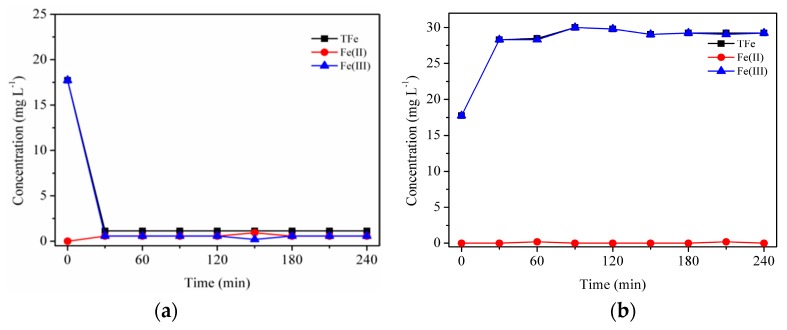
Dissolution kinetics with suspensions of 0.1 g L^−1^ Fhy at pH 3.0 (**a**) under UV illumination and (**b**) in the dark in the presence of 1.0 mM oxalic acid.

By contrast, Fhy dissolution with the assistance of oxalic acid resulted in the production of dissolved TFe with a significant amount (Figure 3). Once Fhy was mingled with oxalic acid at pH 3.0, the aqueous TFe attained 17.73 mg L^−1^, indicating Fhy can be quickly interacted with oxalic acid [26]. When the suspensions were illuminated by UV light, a dramatic decline of aqueous TFe was noticed within the first 0.5 h (Figure 3a), after which it stayed constant at 1.13 mg L^−1^. In addition, pH of the suspensions rose from 3.0 to 6.0 after 4 h of UV illumination. Lan et al. [25] figured out that the aqueous Fe(III)/Fe(II) would all significantly decline in the irradiated systems of goethite and oxalic acid. They owed this observation to the quick jump of pH from 3.5 to 6.0. It can be therefore deduced that a large amount of OH^-^ produced during the light-triggered dissolution of Fhy, which resulted in the hydrolysis of aqueous Fe(III) and the decline of aqueous TFe. However, in the dark, contents of dissolved Fe(III) varied with the opposite trend. It rose to 29.24 mg L^−1^ and remained constant after 120 min reaction, confirming the key role of oxalic acid in promoting the dissolution of Fhy. Aqueous Fe(II) could hardly be found during the total experimental period (Figure 3b), showing that aqueous TFe existed mainly as Fe(III) species. After 240 min reaction, pH value of Fhy suspensions in the dark remained 3.0.

**Figure 4:**
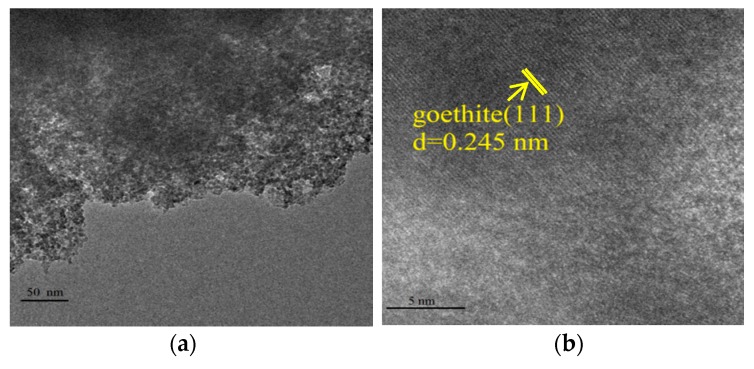
TEM analysis of Fhy (**a**) before and (**b**) after the photodissolution with 1.0 mM oxalic acid.

To better comprehend the photo-triggered dissolution of Fhy with the assistance of oxalic acid, morphological changes of Fhy and newly-produced secondary iron minerals were investigated. XRD analysis of Fhy after 4 h of UV illumination with oxalic acid showed patterns similar to the synthetic 2-line Fhy, suggesting that most of iron oxide remained Fhy (Figure 1). However, an additional new peak at 33.2° was attributed to (130) plane of goethite (JCPDS, No. 810464) [30]. HRTEM image further found (111) lattice fringes with a spacing of 0.245 nm, manifesting the production of goethite in the photo-induced dissolution process of Fhy with oxalic acid, which was in line with the XRD result (Figure 1 and Figure 4).

**Figure 5:**
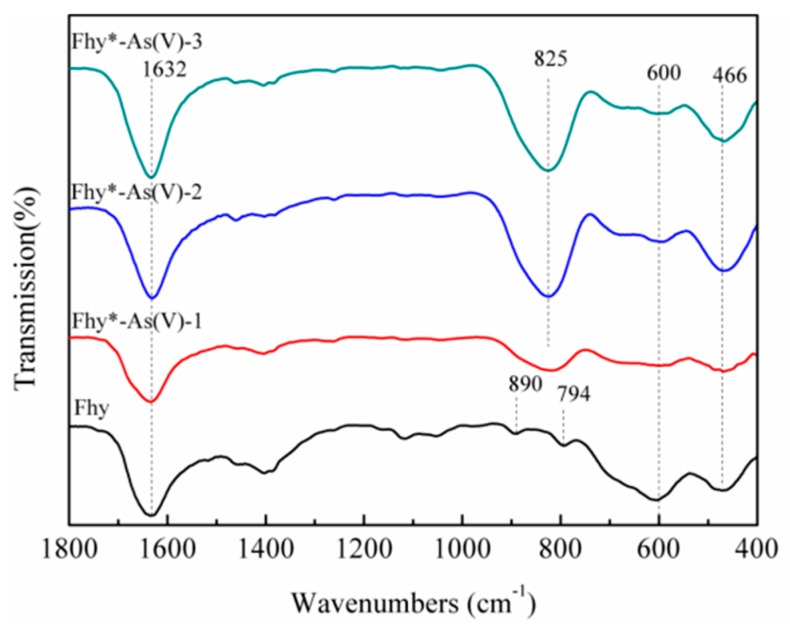
FTIR analysis of Fhy and Fhy*-As(V) after the photodissolution with 1.0 mM oxalic acid.

FTIR analysis of Fhy after 240 min of UV illumination with oxalic acid showed peaks at 794 and 890 cm^−1^ (Figure 5), which is assigned to OH bending mode in goethite [31,32], further verified the transformation of Fhy to goethite. Cornell and Schwertmann [12] suggested that Fhy is metastable and as a precursor to goethite.

### 3.3. Effect of UV/Oxalate on the Dissolution of Fhy*-As(V) and the Mobilization of As(V)

**Figure 6:**
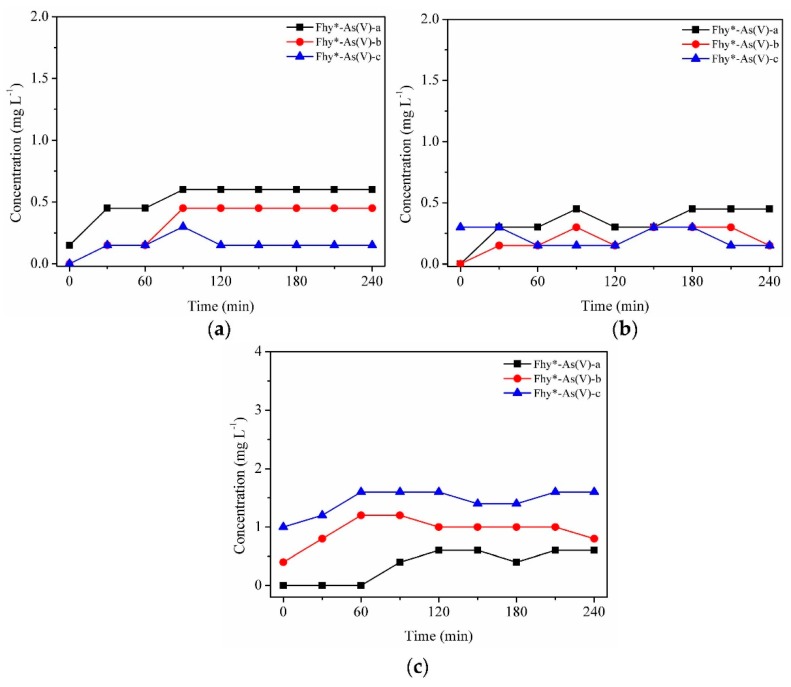
Dissolution kinetics under UV illumination with suspensions of 0.1 g L^−1^ Fhy*-As(V) at pH 3.0. Dissolved TFe (**a**), dissolved Fe(II) (**b**), and As(V) (**c**) were measured.

Obviously, As(V) had an adverse impact on the photodissolution of Fhy. When Fhy*-As(V)-a, Fhy*-As(V)-b and Fhy*-As(V)-c suspensions were illuminated by UV light without the assistance of oxalic acid, the contents of aqueous TFe increased slightly within the first 90 min and then leveled off at 0.60, 0.45, and 0.15 mg L^−1^, respectively (Figure 6a). Dissolved Fe(II) changed in a similar way as the dissolved TFe, and the final concentration reached 0.45, 0.15, and 0.15 mg L^−1^ for Fhy*-As(V)-a, Fhy*-As(V)-b, and Fhy*-As(V)-c systems, respectively (Figure 6b). Nevertheless, nearly no Fhy*-As(V) dissolution was observed in the dark without the assistance of oxalic acid (data not shown).

**Figure 7:**
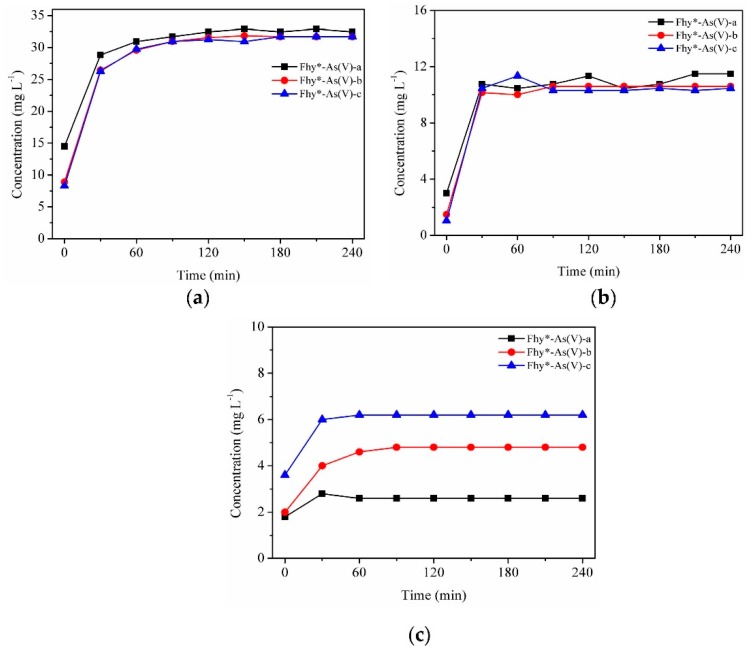
Dissolution kinetics in the dark with suspensions of 0.1 g L^−1^ Fhy*-As(V) at pH 3.0 with 1.0 mM oxalic acid. Dissolved TFe (**a**), dissolved Fe(II) (**b**), and As(V) (**c**) were measured.

The mix of Fhy*-As(V) suspensions and oxalic acid immediately resulted in the occurrence of aqueous TFe. The respective concentrations of TFe were 14.49, 8.91, and 8.30 mg L^−1^ for Fhy*-As(V)-a, Fhy*-As(V)-b, and Fhy*-As(V)-c (Figure 7a). In the dark, both the dissolved TFe and Fe(II) released from Fhy*-As(V) increased first and then leveled off after 3 h reaction. The final contents of aqueous TFe were 32.45, 31.70, and 31.70 mg L^−1^ for Fhy*-As(V)-a, Fhy*-As(V)-b, and Fhy*-As(V)-c systems, respectively (Figure 7a). The change of aqueous Fe(II) was different from the result of Fhy (Figure 6b and Figure 7b). The respective contents of aqueous Fe(II) were 14.49, 8.91, and 8.30 mg L^−1^ for the system of Fhy*-As(V)-a, Fhy*-As(V)-b, and Fhy*-As(V)-c after 4 h reaction.

**Figure 8:**
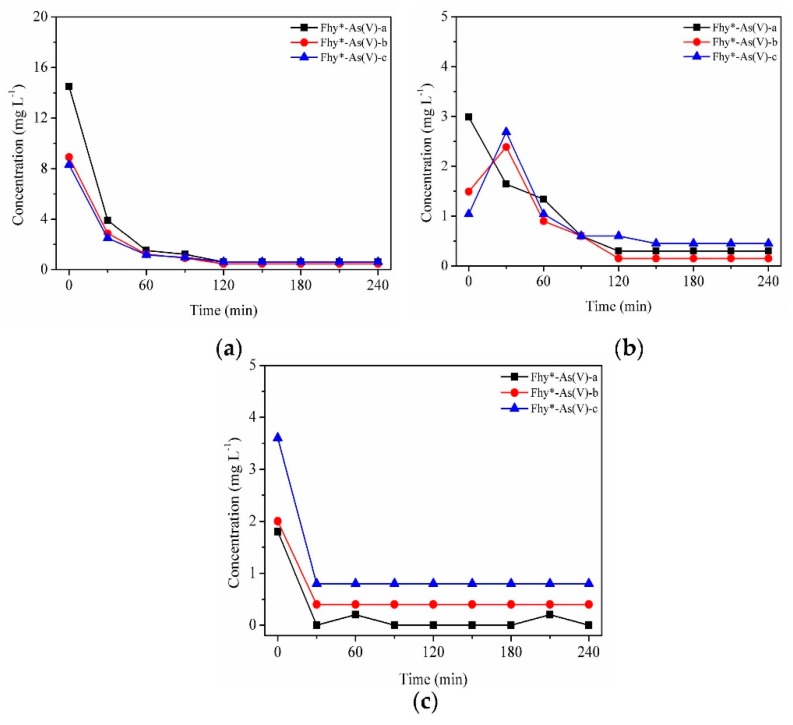
Dissolution kinetics under UV illumination with suspensions of 0.1 g L^−1^ Fhy*-As(V) at pH 3.0 with 1.0 mM oxalic acid. Dissolved TFe (**a**), dissolved Fe(II) (**b**), and As(V) (**c**) were measured.

A downtrend was found for dissolved TFe with the assistance of oxalic acid under UV illumination within the first 120 min, after which it leveled off at 0.60, 0.45, and 0.45 mg L^−1^ for Fhy*-As(V)-a, Fhy*-As(V)-b, and Fhy*-As(V)-c systems, respectively (Figure 8a). It could be interpreted that As(V) occupied surface sorption sites on Fhy and oxalic acid did not touch with Fhy readily, thus caused less dissolution of Fhy*-As(V) [33]. In Fhy*-As(V)-a, aqueous Fe(II) changed in a similar trend to aqueous TFe. While for Fhy*-As(V)-b and Fhy*-As(V)-c, dissolved Fe(II) contents lifted to 2.39 and 2.69 mg L^−1^ in the first 0.5 h, respectively. Afterwards, the concentration of dissolved Fe(II) had a sink of about 2.30 mg L^−1^ between 0.5 h and 2.0 h (Figure 8b).

As(V) fate during the photodissolution of Fhy*-As(V) without the assistance of oxalic acid was presented in Figure 6c. For Fhy*-As(V)-a, Fhy*-As(V)-b, and Fhy*-As(V)-c systems, As(V) contents rose and attained the peak values at 0.6, 1.0, and 1.6 mg L^−1^ in the first 2 h of UV illumination, respectively. With the assistance of oxalic acid, two stages were found for the migration of As(V) (Figure 7c and Figure 8c). The released As(V) into solution was 1.8, 2.0, and 3.6 mg L^−1^ after the mix of oxalic acid and Fhy*-As(V) suspensions for Fhy*-As(V)-a, Fhy*-As(V)-b, and Fhy*-As(V)-c, respectively. Oxalic acid complexed with Fe atoms and dragged them out from the surface of Fhy, and As(V) would not be liberated until the Fe atoms that held As(V) were dissolved. From Figure 7c and Figure 8c, the mobilized As(V) leveled off after 30 min reaction both under UV irradiation and in the dark, which is attributed to the rapid reaction between oxalic acid and Fhy*-As(V). However, they had a different change trend in the first 30 min, with the aqueous As(V) increase in the dark and decrease under UV illumination. The final contents of As(V) in the dark were 2.6, 4.8, and 6.2 mg L^−1^, 7.75−13.0 times as that under UV illumination, for Fhy*-As(V)-a, Fhy*-As(V)-b, and Fhy*-As(V)-c systems, respectively (Figure 7c and Figure 8c). A pH increases of the suspension from 3.0 to ~6.0 was found in the photodissolution process of Fhy*-As(V) with oxalic acid. Fe(III) hydrolysis at high pH might result in the generation of goethite, which was conducive to the re-sorption of aqueous As(V). As a result, oxalic acid only resulted in 0.2, 0.4, and 0.8 mg L^−1^ mobilized As(V) under UV light illumination for Fhy*-As(V)-a, Fhy*-As(V)-b, and Fhy*-As(V)-c systems, respectively (Figure 8c).

To clarify the composition changes of iron mineral during the dissolution of Fhy*-As(V), XRD analysis was conducted. However, no other peaks could be observed from XRD results of Fhy*-As(V) after 4 h of UV illumination with the assistance of oxalic acid, probably due to the suppression of As(V) on the transformation of Fhy or low contents of newly-formed iron minerals. A previous study suggested that As(V) adsorbed on Fhy significantly inhibited the transformation of Fhy to goethite as well [27].

### 3.4. Mechanism of Photodissolution and As(V) Fate in the System of Fhy*-As(V)

**Figure 9:**
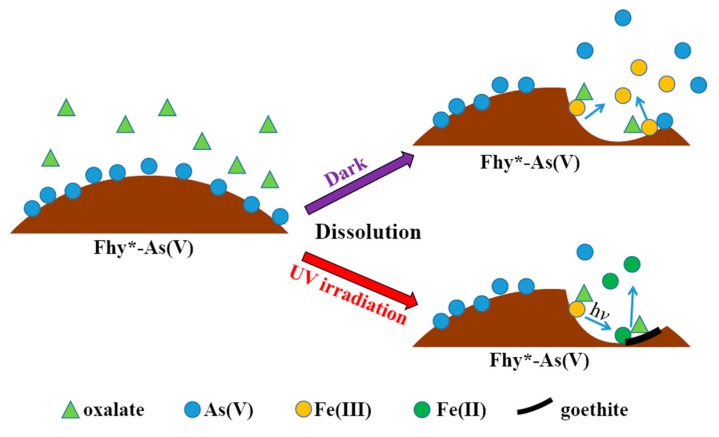
Proposed mechanisms of Fhy photodissolution and As(V) migration in Fhy*-As(V).

On account of the above points and published works [7,18,23,26,27], a possible mechanism of Fhy dissolution triggered by oxalic acid and As(V) migration in Fhy*-As(V) with and without UV light is illuminated (Figure 9). Oxalic acid firstly forms ${\left[\equiv {\mathrm{Fe}}^{\mathrm{III}}{\left(C_{2}O_{4}\right)}_{n}\right]}^{\left(2n-3\right)-}$ complexes with Fe(III) on the surface of Fhy via sorption interaction [Equation (1)]. Herein, the symbol ≡ represents the surface site of an iron (hydr)oxide. Subsequently, ${\left[\equiv {\mathrm{Fe}}^{\mathrm{III}}{\left(C_{2}O_{4}\right)}_{n}\right]}^{\left(2n-3\right)-}$ is excited by UV light to produce surface Fe(II) complexes of ${\left[\equiv {\mathrm{Fe}}^{\mathrm{II}}{\left(C_{2}O_{4}\right)}_{\left(n-1\right)}\right]}^{4-2n}$ and some radicals containing oxalic acid radical [${\left(C_{2}O_{4}\right)}^{•-}$], carbon dioxide anion radical [${\left({\mathrm{CO}}_{2}\right)}^{•-}$], and superoxide radical ($O_{2}{}^{•-}$) [Equations (2–4)]. $O_{2}{}^{•-}$ can further react with Fe^3+^ to generate Fe^2+^ and O_2_ [Equation (5)]. Fe(III)-oxalic acid complexes of ${\left[{\mathrm{Fe}}^{\mathrm{III}}{\left(C_{2}O_{4}\right)}_{n}\right]}^{3-2n}\;$in the suspension could be reduced as well under UV illumination according to Equation (6). Without the assistance of UV light, the adsorbed As(V) on Fhy releases into the solution during the dissolution process, with the peak As(V) concentration of 6.2 mg L^−1^ (Figure 7c). However, with the assistance of UV light, As(V) migration in the Fhy*-As(V) system is inhibited apparently [the peak As(V) concentration is only 0.8 mg L^−1^ (Figure 8c)], which might be ascribed to some liberated As(V) in suspension being re-fixed on residual Fhy and newly-produced goethite [27]. As a result, the effect of UV light should be considered when evaluating the fate of As(V) in water environments including iron minerals and organic acids.

(1) $$\mathrm{Fhy}\;+\;n{\;H}_{2}C_{2}O_{4}\;↔{\left[\equiv {\mathrm{Fe}}^{\mathrm{III}}{{(C}_{2}O_{4})}_{n}\right]}^{(2n-3)-}\;$$

(2) $${\left[\equiv {\mathrm{Fe}}^{\mathrm{III}}{{(C}_{2}O_{4})}_{n}\right]}^{(2n-3)-}\;+\;hv\;\to \;[\;\equiv {\mathrm{Fe}}^{\mathrm{II}}{{(C}_{2}O_{4})}_{\left(n-1\right)}{]}^{4-2n}{\;+\;C}_{2}O_{4}{}^{•-\;}$$

(3) $$C_{2}O_{4}{}^{•-\;}\;\to {\;\mathrm{CO}}_{2}{\;+\;\mathrm{CO}}_{2}{}^{•-}$$

(4) $${\mathrm{CO}}_{2}{}^{•-}{\;+\;O}_{2}\;\to {\;\mathrm{CO}}_{2}{\;+\;O}_{2}{}^{•-}$$

(5) $$O_{2}{}^{•-}\;+{\;\mathrm{Fe}}^{3+}\;\to {\;\mathrm{Fe}}^{2+}\;+{\;O}_{2}$$

(6) $${{[\mathrm{Fe}}^{\mathrm{III}}{{(C}_{2}O_{4})}_{n}]}^{3-2n}\;+\;hv\;\to {\;[\mathrm{Fe}}^{\mathrm{II}}{{(C}_{2}O_{4})}_{\left(n-1\right)}{]}^{4-2n}{\;+\;C}_{2}O_{4}{}^{•-}$$

## 4. Conclusions

This study investigates the impacts of oxalic acid and UV light illumination on the dissolution of naked Fhy and Fhy loaded with As(V). In the dark and under UV illumination, roles of oxalic acid are different for the dissolution of Fhy. Fhy dissolution triggered by oxalic acid was principally mediated by the ligand-facilitated process in the dark. Complexes of Fe(III)-oxalic acid produced on Fhy were transformed into Fe(II)-oxalic acid by UV light. For Fhy*-As(V) systems, UV illumination has almost no impact on the migration of As(V) without the existence of oxalic acid. Nevertheless, with the assistance of oxalic acid, UV light illumination resulted in the released As(V) contents decreased by 775%−1300% compared to that in the dark. Considering the coexistence of As(V), oxalic acid and iron oxides in acidic water environments, this study revealed that solar light (containing UV) illumination could improve the retention of As(V) on Fhy in the aquatic environments including oxalic acid.

## Figures and Tables

**Table 1:** The loaded As(V) amounts on Fhy at pH 3.0.

No.	As(V) Loading (mg g^−1^)
Fhy*-As(V)-a	99.9
Fhy*-As(V)-b	146.8
Fhy*-As(V)-c	196.8
